# Natural variation among *Arabidopsis thaliana* accessions in tolerance to high magnesium supply

**DOI:** 10.1038/s41598-018-31950-0

**Published:** 2018-09-11

**Authors:** Yaofang Niu, Ping Chen, Yu Zhang, Zhongwei Wang, Shikai Hu, Gulei Jin, Caixian Tang, Longbiao Guo

**Affiliations:** 1 State Key Laboratory of Rice Biology, China National Rice Research Institute, Hangzhou, 310006 China; 2 College of Agronomy and Biotechnology, Zhejiang University, Hangzhou, 310029 China; 3 Centre for AgriBioscience, La Trobe University, Melbourne Campus, Vic, 3086 Australia

## Abstract

High magnesium (Mg^2+^) in some extreme serpentine soils or semi-arid regions is an important factor affecting crop growth and development. Specific loci that form the genetic framework underlying high Mg^2+^ homeostasis, however, are not well understood. By using GWA mapping on 388 accessions of *Arabidopsis thaliana* selected from a worldwide collection and genotyped at approximately 250,00 SNPs, we successfully identified 109 and 74 putative genetic regions associated in nutrient traits under normal (1,000 µM) and high Mg^2+^ (10,000 µM), respectively. Above 90% SNPs associated with nutrients including Mg^2+^ and only two SNPs shared between normal and high Mg^2+^. A single strong peak of SNPs associated with Ca concentration corresponding to candidate gene *At1g604*2*0 ARABIDOPSIS NUCLEOREDOXIN* (*AtNRX1*) under high Mg^2+^ was further determined. Compared with wildtype, mutants of *Atnrx1-1* and *Atnrx1-*2 supplied with high Mg^2+^ had higher Ca concentrations in the plant, and higher cytosolic Ca^2+^ concentrations during root elongation, as well as higher fresh weight and lateral-root number. This suggests that *AtNRX1* was a critical gene negatively regulating Ca uptake under high Mg^2+^ conditions. The discovery could help to breed/select crops that can adapt to high-Mg^2+^ soils such as serpentine soils (high ratio of Mg^2+^: Ca^2+^) or Mars soil with high levels of magnesium sulfate.

## Introduction

Magnesium (Mg) is the 8^th^ most abundant mineral element on earth and the fourth abundant mineral element in plants following nitrogen (N), potassium (K) and calcium (Ca)^1^. Magnesium in soils originates from source rock material containing various types of silicates and carbonates. In extreme serpentine (high magnesium: calcium) soils^2^ and in semi-arid regions, where water stress conditions^3^ can lead to Mg accumulation. In addition, Mars regolith (unconsolidated surface material) is a potential medium for plant growth in bioregenerative life support systems. However, analyses by the Mars Exploration Rover Landers at Meridiani Planum and Gusev Crater have also suggested there are particularly high levels of magnesium sulfate minerals in outcrops and soils^4–6^.

Although plants rely on a sufficient supply of Mg^2+^ for normal growth and development, excessive Mg^2+^ accumulation often causes toxicity to plant cells. Plants grown in high-Mg^2+^ soils like serpentine or Mars soils may alleviate Mg toxicity by limiting internal Mg accumulation, Mg excretion from leaves and/or increasing plant tolerance to high Mg^2+^ concentrations via altering leaf size, sclerophylls, stature and root systems^7,8^. Previous reciprocal transplant experiments between serpentine populations (or species) and their spatially adjacent populations (or sister species) growing on serpentine soils demonstrated unequivocally that adaptation to serpentine soils had a genetic basis^9–12^. Several quantitative trait loci that control contrasting adaptive traits in serpentine-tolerant *Microseris douglasii* and serpentine-intolerant *M. bigelovii* have also been identified^13^. A high degree of polymorphism has been reported among plant species in terms of growth tolerance to serpentine soils^14–16^.

This present study employed genome-wide association studies (GWAS), an approach that has been applied successfully to identify many different traits ranging from genetically simple traits to more complex features^17^, to identify candidate genes likely to be involved in adaptation to high Mg^2+^, based on 388 accessions of *Arabidopsis thaliana* selected from a worldwide collection. *Arabidopsis thaliana* has a broad geographical distribution and consequently is subject to varying nutritional environments, which makes it a useful model species for studying nutrient homeostasis^18,19^ in a wide variety of environments^20^. Importantly, more than 1300 distinct accessions have been genotyped^21^ and to date more than 1000 inbred lines have been ‘fully sequenced’. Meanwhile, automated phenotyping platforms have been developed^22–24^, allowing for precise and high-throughput measurements of plant growth. These developments enhance our ability to map causal genetic polymorphisms through GWAS. Moreover, high Mg^2+^ decreased the growth of *Arabidopsis* including biomass production, root hair development and the absorption of other nutrients^25–28^. To further identify the likely candidate genes that are involved in adaptation to high-Mg^2+^ soils, we used a chemically-defined nutrient solution that contained 10,000 μM MgSO_4_ of maximum authentic high Mg^2+^ soils to perform a viability screen on a large collection of wild-type accessions of *Arabidopsis thaliana*. This study aimed to (1) investigate natural variations in growth response to high Mg^2+^, and (2) identify novel genes tolerant to Mg^2+^ excess.

## Materials and Methods

### Plant material and growth conditions

Seeds of 388 accessions with accessions CS76636, CS76427, CS78885 and CS22660 and two T-DNA insertion lines SALK_100357C and SALK_113401C were derived from the *Arabidopsis* Biological Resources Center stock center (http://nasc.nott.ac.uk). The accessions were selected without any *a priori* consideration of their natural habitat, including geographical distribution or precipitation levels, and were collected from the wild and available in the stock center or soon-to-be-released collections. Most of these accessions were originated from Europe including Sweden, Germany, France, Czech Republic and United Kingdom, and from North America and Middle Asia, representing the sampling strategy of the 1,307 worldwide accessions^21^. This was consistent with the distribution of the 1,307 *Arabidopsis* lines collected in RegPanel.

### Arabidopsis cultivation

The 388 accessions of *Arabidopsis thaliana* were grown under 2 Mg^2+^ treatments, which were achieved by altering the concentration of MgSO_4_ in the basal medium. To minimise maternal effects prior to phenotyping, natural accessions were grown for one generation during 2015 under controlled greenhouse conditions at Zhejiang University (N30°18′25, E120°04′54). For surface sterilization, seeds of various accessions were placed for 1 h in open 200-μL PCR tubes in a sealed box producing chlorine gas generated from 13 mL of 10% sodium hypochlorite and 350 μL of 37% hydrochloric acid. Sterile seeds were then put on the surface of 30 mL agar media, containing 1.2% (w/v) agar and 0.6% (w/v) sucrose (A-1296; Sigma-Aldrich; http://www.sigmaaldrich.com) in 10 × 10-cm^2^ plates with grid schematic engraved below the plate. Plates were positioned in racks and oriented in a vertical position, and were kept at 4 °C for 48 h in the dark for seed stratification. Thereafter, the racks containing the plates were transferred to a growth chamber under a 10-h light/14-h dark photoperiod at constant temperature of 22 °C, 60% relative humidity and light intensity of 120 µmol photons m^−2^ s^−1^. Twelve seedlings were grown in each plate and each treatment received at least four independent replicates. The racks were removed to the image acquisition room once per day and then immediately returned to the growth chamber. Throughout the experiments, the plate position within the box and box position in the growth chamber were re-randomized daily. For assays on agar plates, studies were performed on 8-d-old plants; that is, at an early stage of their stability and homogeneity growth phase. Moreover, many previous publications adopted 6- to 8-d-old *Arabidopsis* seedlings under stresses for determining *Arabidopsis* morphology, physiology, and development as well as responses to nutrient stresses^29–31^.

### Mg^2+^ Treatments

The basal medium (Normal Mg^2+^, N), which was used as control, contained (µM) 1500 KNO_3_, 500 NaH_2_PO_4_, 1000 CaCl_2_, 250 (NH_4_)_2_SO_4_, 1000 MgSO_4_, 25 Fe-EDTA, 10 H_3_BO_3_, 0.5 MnSO_4_, 0.5 ZnSO_4_, 0.1 CuSO_4_ and 0.1 (NH_4_)_6_Mo_7_O_24_. The normal and high Mg^2+^ treatments contained 1,000, and 10,000 µM MgSO_4_, respectively, which was in accordance with Mg^2+^ concentrations in soil solutions^27^. 10,000 μM Mg^2+^was selected in the present study for the quantitative analysis of the natural variation of *Arabidopsis* in response to high Mg^2+^ stress because this concentration had an adverse effect on growth without toxic symptoms of most natural accessions^27,32^. Besides, the concentrations of Mg^2+^ in the control were 1000 µM, that has been adopted for *Arabidopsis* growth by many plant biologists^33–35^. Though the concentration of SO_4_^2−^ increased 10 times comparing with that in the normal medium, SO_4_^2−^ had hardly affected morphogenesis of *Arabidopsis*^27,36^. The pH of the growing media was adjusted to pH 5.8 with MES (N-morpholino) ethane-sulphonic acid)-KOH buffer before autoclaving.

### Phenotype analysis

Each line had four biological replicates for each treatment. Seeds that had not germinated at day 6 were discarded from further analysis, resulting in approximately 3–4 seedlings analyzed per genotype per treatment. The 21 traits under each treatment were classified into 2 categories: 7 morphological traits and 14 nutrient traits. Seedlings were photographed with a high-resolution digital camera (Sony RX100, Japan) daily for determination of root and shoot growth. Root or shoot biomass, and the number of days from seeding to emergence (>50% seedlings having the first radicle or cotyledon), were recorded. Meanwhile, photographs after 8 days of treatment were analyzed and quantified for phenotype using the public domain image analysis program Image J version 1.43 (http://rsb.info.nih.gov/ij/)^27^. The length of primary roots, rosette diameter and epicotyl were determined across the median seedling using Image J. Lateral-root number was determined by counting the number of true roots (>1 mm long lateral root primordia) per primary root. For each condition, an independent sample accession consisted of four plants at the same growth stage, and the time of sampling was the end of the light period of day 8.

### Analyses of mineral homeostasis

After 8-d growth at various Mg^2+^ concentrations, plants were harvested. All fully-expanded seedlings without lesions were collected, and weighed. The results were the average value across all available replicates. Seedlings were washed thoroughly with ultrapure water and dried in an oven at 75 °C for 24 h. The dried root and shoot samples were then wet-digested in the concentrated HNO_3_/H_2_O_2_ at 90, 120 and 140 °C for 2 h, respectively, until there was no brown fume, and then further digested at 180 °C until the digest became clear. Concentrations of potassium (K), calcium (Ca), Magnesium (Mg), sulfur (S), iron (Fe), manganese (Mn) and sodium (Na) in the digests were analyzed by ICP-MS (Inductively coupled plasma mass spectrometer, Agilent 7500a, USA), the contents per plant were calculated. Mineral analysis was done in the whole*-*plant.

### Fluo 4-AM loading and confocal imaging of cytosolic Ca^2+^ concentration in root

The Ca^2+^-sensitive fluorescent dye 1-[2-amino-5-(2,7-difluoro-6-hydroxy-3-oxo-9-xanthenyl)phenoxy] -2-(2-amino-5-methylphenoxy)ethane-N,N,N′,N′-tetraacetic acid, pentaacetoxymethyl ester (Fluo-4/AM) ester was used to estimate cytosolic Ca^2+^ concentration in the roots. The Fluo-4/AM ester was loaded into the root at 37 °C in the dark at a final concentration of 5 *μ*M Fluo 4-AM dye and Pluronic F-127 (0.05%) in the Hank’s Balanced Salt Solution (HBSS) solution. After 1-h incubation, the roots were washed with the standard HBSS medium three times and left at 25 °C for 30 min. The samples were mounted and photographed with a Zeiss LSM 780 (Zeiss Co., Germany). Fluorescence was detected using 488 nm excitation and 505–545 nm band pass filter. Roots including root hairs were placed on the stage of a Zeiss Axiovert inverted microscope attached to a LSM780 laser scanning confocal microscope, and imaged using a Zeiss 20×, N.A., wet objective. Each frame represents a 7-s scan of the laser.

### Genome-Wide Association (GWA) mapping, SNP selection, and statistics

The GWA mapping was performed on the values of epicotyl length, rosette width, primary root length, and lateral root number of the median seedling of each accession. For traits of and nutrients, means values of 4 biological replicates were used. We linked 21 traits under normal or high Mg^2+^ (Supporting Information Table S1) to published genomic data on accessions from a 250 K SNP chip. The GWA analysis was performed in the GWAPP web (http://gwas.gmi.oeaw.ac.at/) interface using the mixed model algorithm method^37^. A kinship matrix was generated using the identity in state of SNPs between each pair of accessions^38^. The *P*-value above bias due to population stratification was evaluated with Q–Q plots. Candidate loci were selected based on the minor allele frequency, LOD score and trait-associated SNPs and their position in the genome to pinpoint candidate genes within 5 kb up- and down-stream of the identified SNP (Supporting Information Table S2). The putative candidate genes corresponding to each SNP are listed in Supporting Information Table S3. For each of the candidate genes, the annotations were retrieved from TAIR10 (*Arabidopsis*.org).

### Extraction of total RNA and quantitative PCR

Samples were collected from 8-day-old *Arabidopsis* after grown at normal and high Mg^2+^ treatments. For each condition, four samples were taken on independent seedling materials from eight different plants at the same growth stage and the time of sampling was the end of the light period of day 8. Total DNA was extracted by Plant DNA Isolation Reagent (Takara) from about 80 mg of fresh seedling tissues of four independent biological replicates. Two homozygous T-DNA insertion lines (SALK_100357C, *Atnrx1-1*) and (SALK_113401C, *Atnrx1-*2) was conducted by PCR with gene-specific primers SALK_100357C LP: 5′-AACCCAACCATCTTTGGACTC-3′; SALK_100357C RP: 5′-TCAAGACTTCAAGACCAAGCC-3′ and T-DNA border primer LBb1.3:5′-ATTTTGCCGATTTCGGAAC-3′; SALK_113401C, LP: 5′-ATCCACTTTTGTCGTTGAACG-3′; SALK_113401C RP: 5′-CGATCGCAACTTCTTCTGATC-3′ and T-DNA border primer LBb1.3:5′-ATTTTGCCGATTTCGGAAC-3′; Total RNA was extracted by RNAisoPlus (Takara, Otsu, Shiga, Japan) from about 70 mg of fresh seedling tissues of four independent biological replicates. All RNA samples were checked for DNA contamination before cDNA synthesis. cDNA was synthesized, and possible residual genomic DNA contamination was verified as described in our previous study^39^. The mRNA levels of *AT1G60420* in two lines of SALK_100357C and SALK_113401C were detected by the SYBR Green RT-PCR kit (Takara) with following pairs of gene-specific primers: *AT1G60420*, fw: 5′- TTCTCGGCCCTGATGGAAAAACC-3′, rv: 5′- GCTTCATTCCGCTCCTTTATCTGCT-3′ A pair of housekeeping gene of *UBQ10* (fw: 5′-GGTTCGTACCTTTGTCCAAGCA-3′, rv: 5′-CCTTCGTTAAACCAAGCTCAGTATC-3′) was used for a control PCR. Melting curve analysis and gel electrophoresis of the PCR products were used to confirm the absence of non-specific amplification products. Relative expression levels were calculated by subtracting the threshold cycle (Ct) values for *UBQ10* from those of the target gene (to give ∆Ct) and then calculating 2^−∆Ct^, where C_t_ refers to the cycle number at which the fluorescence rises above the set threshold qPCR. *UBQ10* was chosen as the housekeeping reference^40^.

### Phylogenetic and Statistical Analyses

DNA sequences were obtained from the 1001 Genomes project (www.1001genomes.org). All statistical analyses were conducted with DPS software (Stirling Technologies Inc., China). Means were compared by the t test or the Fisher’s least significant difference test at *P* = 0.05.

## Results

### Distribution and correlation of different 21 traits under normal and high Mg^2+^

**Figure 1:**
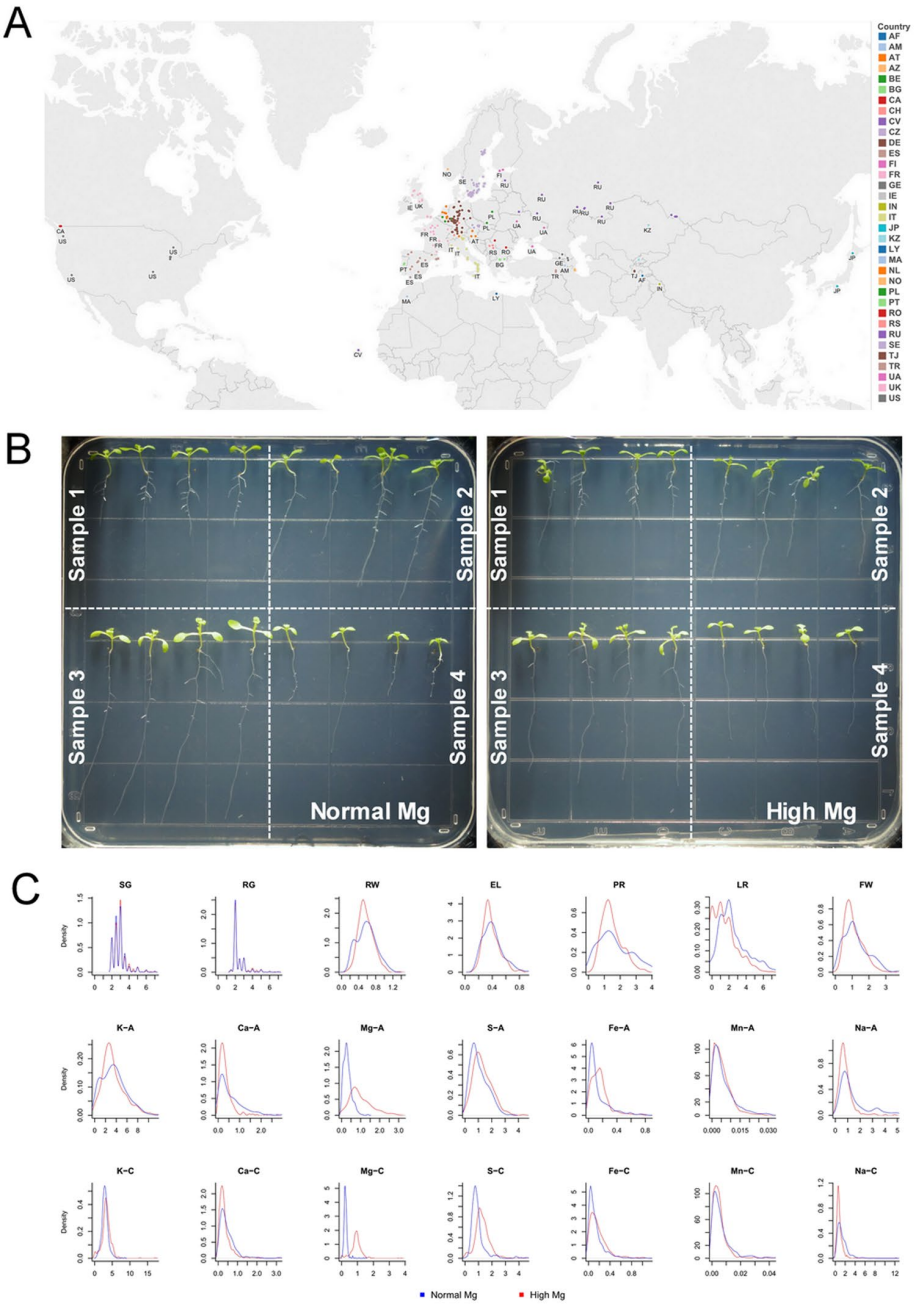
Worldwide diversity of 388 *A. thaliana* natural accessions and cultivation used in this study according to their reported country origins and frequency distributions of non-normalized data of all traits. (**A**) The data were exported for visualization and geographical mapping in Tableau Desktop 8.1 (http://www.tableausoftware.com/). (**B**) Cultivation for 388 *Arabidopsis* inbred lines at Zhejiang University, Hangzhou, China. Each line was treated with low (1 µM), normal (1,000 µM), and high Mg^2+^ (10,000 µM). Each line had four biological replicates, and the median value was used in the analysis. (**C**) Frequency distributions of non-normalized data of all traits in the 388 population. Blue and Red line indicates normal and high Mg^2+^, respectively.

Distribution of 388 *Arabidopsis* accessions collected from the wild and available in the stock center or soon-to-be-released collections is shown in Fig. 1A. In general, high Mg^2+^ supply (10,000 μM) had an adverse effect on the growth of most natural accessions. This high Mg^2+^ concentration induced a large degree of phenotypic variation and thus was selected for the quantitative analysis of the natural variation of *Arabidopsis* in response to high Mg^2+^ stress (Fig. 1B). A subset of 388 accessions (250 K SNPs) was characterized phenotypically for multiple traits, including root emergence (RG), shoot emergence (SG), primary root length (PR), lateral root number (LR), epicotyl length (EL), rosette width (WD), fresh weight (FW) of the plants and fourteen nutrient traits, including absorption per plant and concentration of K, Ca, Mg, S, Fe, Mn and Na under normal and high Mg^2+^ supplies (see Table 1 and ‘*Phenotype analysis’* in Materials and Methods).

**Table 1:** Summary for 21 *Arabidopsis* traits.

Traits	Full name of the trait	Unit	Trait description
RG	Days to root germination	day	The number of days from seeding until emergence, with more than half of the seedlings having a first radicle.
PR	Primary root length	cm	After 8 days of growth under the two treatment conditions, the plants were flattened directly on agar and imaged using a camera. The primary root lengths and lateral root number were determined across the median seedling using ImageJ.
LR	Lateral root number		
SG	Days to seed germination	day	The number of days from seeding until emergence, with more than half of the seedlings having the first cotyledon.
EL	Epicotyl length	cm	After 8 days of growth under the two treatment conditions, the plants were flattened directly on agar and imaged using a camera. The epicotyl length and rosette width were determined across the median seedling using ImageJ.
RW	Rosette width	cm	
FW	Fresh weight of plants	mg	All fully-expanded seedlings without lesions were collected from each of 4 plants for each accession and weighed to obtain the fresh weight. The results are the average value across all available replicates.
K-A	The amount of potassium absorbed per plant	mg K/plant	Elemental analysis was performed with an ICP-MS (Agilent 7500a, USA). All samples were normalized to calculated weights as previously described. The ‘Nutrient absorption(A)’ was expressed as total amount of nutrient per plant; ‘Nutrient concentration (C)’ was calculated by total amount of nutrient per plant /plant fresh weight (FW). The results are the average value across all available replicates.
K-C	Potassium concentration per fresh weight	mg K/g (FW)	
Ca-A	The amount of calcium absorbed per plant	mg Ca/plant	
Ca-C	Calcium concentration per fresh weight	mg Ca/g (FW)	
Mg-A	The amount of magnesium absorbed per plant	mg Mg/plant	
Mg-C	Magnesium concentration per fresh weight	mg Mg/g (FW)	
S-A	The amount of sulfur absorbed per plant	mg S/plant	
S-C	Sulfur concentration per fresh weight	mg S/g (FW)	
Fe-A	The amount of iron absorbed per plant	mg Fe/plant	
Fe-C	Iron concentration per fresh weight	mg Fe/g (FW)	
Mn-A	The amount of manganese absorbed per plant	mg Mn/plant	
Mn-C	Manganese concentration per fresh weight	mg Mn/g (FW)	
Na-A	The amount of sodium absorbed per plant	mg Na/plant	
Na-C	Sodium concentration per fresh weight	mg Na/g (FW)	

Before performing the quantitative genetic analyses, we assessed the distributions of all traits based on all raw data (Supporting Information Table S1). The phenotypic analysis of the 388 accessions showed that the frequency distribution of all traits was close to normality (Fig. 1C; Table 1). Further detailed analysis showed that distribution of Mg-A-H (Mg^2+^ absorption under high Mg^2+^) and Mg-C-H (Mg^2+^ concentration under high Mg^2+^) showed a great right-skewed probability density with the right-skewed being greater for Mg-C-H than that for Mg-A-H, suggesting that high Mg^2+^ supply increased Mg concentration of most *Arabidopsis* accessions at least in the early phase of development. However, only slight skewness and kurtosis was observed for S absorption and concentration (Fig. 1C). Distribution of RW, EL, PR, FW, Ca-A (Ca absorption), Ca-C (Ca concentration), Na-A (Na absorption) and Na-C (Na concentration) presented a standard normal distribution but with a sharp peak upon high Mg^2+^ as compared with normal Mg^2+^ (Fig. 1C), indicating that the high Mg^2+^ treatment resulted in a more focused response in those traits. Notably, the right side of the curve of absorption and concentration of Ca, Fe and Mn under both normal and high Mg^2+^ fitted a normal distribution, but the left-hand side deviated significantly from the normal distribution, displaying a decapitation tail (Fig. 1C).

**Figure 2:**
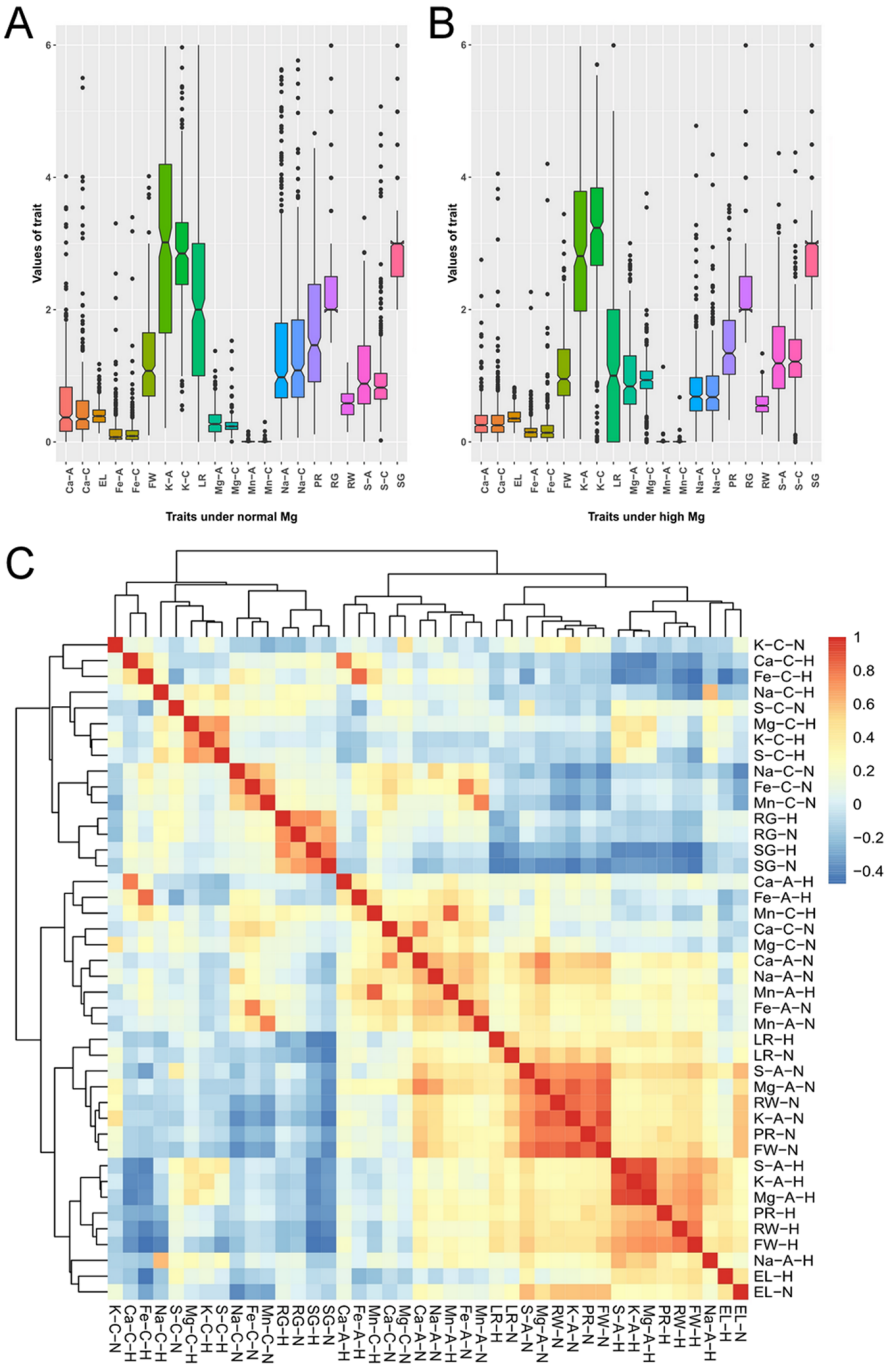
Natural variation in traits of *Arabidopsis* accessions grown at normal (1,000 µM) (**A**) and high Mg^2+^ (10,000 µM). (**B**) The average value per accession was calculated, and values of individual traits were plotted using the ‘ggplot2’ package in R. Accessions representing outliers for a trait are indicated by closed circles. (**C**) Heat map represents cluster of the pearson correlation coefficient between each pair of 42 phenotypes, the pearson’s correlation coefficients were normalized (z-score) and represented with blue-red color scheme.

Data of the traits are close to normal distribution reflecting the homogeneity of the investigated population. However, what was the response of each individual domain to high Mg^2+^? According to the date (Supporting Information Table S1), the effects of high Mg^2+^ on all phenotypes can be divided into two types. First, high Mg^2+^ had a similar promoting effect on LR, Mg-A, Mg-C, S-A and S-C of most accessions. Second, high Mg^2+^ enhanced traits of PR, EL, RW, FW and absorption and concentrations of K, Ca, Fe and Mn in the accessions with low value while reduced them in the accessions with high value as compared with normal Mg^2+^ supply. Furthermore, the initial comparison of 21 plant traits between normal and high Mg^2+^ was performed using statistical analysis by a paired two-tailed Student t test, with a *P*-value < 0.01 (Table 2). Accordingly, the above first type traits were consistently changed by high Mg^2+^ as compared with normal Mg^2+^. Apart from this, the above second traits were inconsistently altered by high Mg^2+^ (Table 2, Fig. 2A,B).

**Table 2:** Minimum (Min), maximum (Max), mean and standard deviation (SD) values of individual traits for 21 phenotypes under high (10,000 µM) and normal Mg^2+^ (1,000 µM).The correlations (r^2^) between values of individual traits of the plants grown at high and normal Mg^2+^ are also shown.

Normal Mg^2+^	Min	Max	Mean	SD	*p*-value (normality test)^a^		
RG	1.5000	7.0000	2.4000	0.8200	1.07E-28		
PR	0.1155	4.6749	1.6712	1.0430	4.61E-10		
LR	0	9.0000	2.4845	1.7004	2.00E-16		
SG	2.0000	7.0000	2.8814	0.7949	6.07E-22		
EL	0.1305	1.1846	0.4088	0.1590	1.73E-12		
RW	0.1493	1.2021	0.5823	0.2241	7.13E-05		
FW	0.1000	4.025	1.2242	0.7383	3.26E-11		
K-A	0.2105	14.185	3.6072	2.3203	8.73E-11		
K-C	0.4935	8.5100	2.9159	0.9256	2.17E-11		
Ca-A	0.0010	4.0225	0.5932	0.6245	2.49E-22		
Ca-C	0.0009	7.4220	0.5646	0.8206	1.01E-31		
Mg-A	0.0033	1.3830	0.3042	0.2049	6.52E-13		
Mg-C	0.0118	1.5367	0.2547	0.1369	1.50E-27		
S-A	0.0030	3.3967	1.0466	0.6320	1.62E-10		
S-C	0.0300	6.7483	0.9736	0.6864	1.85E-28		
Fe-A	0.0008	3.3113	0.1664	0.2977	1.45E-32		
Fe-C	0.0007	8.7300	0.1826	0.5313	4.24E-37		
Mn-A	0.0000	0.1647	0.0075	0.0134	2.73E-33		
Mn-C	0.0000	0.3088	0.0084	0.0201	7.84E-36		
Na-A	0.0300	5.6400	1.4760	1.2287	2.86E-21		
Na-C	0.0643	21.200	1.4744	1.6282	3.50E-31		
**High Mg** ^**2+**^	**Min**	**Max**	**Mean**	**SD**	***p*** **-value (normality test)** ^**a**^	**r** ^**2**^ **(H vs N)**	***p*** **-value (t-test)** ^**b**^
RG	1.5000	7.0000	2.4526	0.8379	2.54E-27	0.53	0.3607
PR	0.3301	3.5816	1.4777	0.6374	5.06E-10	0.3115	0.0019
LR	0.0000	7.0000	1.4845	1.3780	2.51E-17	0.4146	1.86E-18
SG	2.0000	7.0000	2.9021	0.7731	2.05E-20	0.5337	0.7143
EL	0.1327	0.8300	0.3825	0.1190	6.46E-10	0.2886	0.0092
RW	0.1111	1.3434	0.5714	0.1754	0.000113	0.2034	0.4492
FW	0.0500	3.4500	1.0945	0.5354	4.64E-12	0.3430	0.0052
K-A	0.0413	12.0925	3.4778	2.0909	2.89E-12	0.1070	0.4147
K-C	0.0148	17.2500	3.3030	1.4688	1.05E-20	0.0102	1.3130
Ca-A	0.0003	22.1050	0.3740	1.1442	1.54E-38	0.0057	0.0010
Ca-C	0.0002	36.8417	0.4576	1.9330	5.79E-39	0.0023	0.3163
Mg-A	0.0048	3.0103	0.9847	0.5822	2.78E-11	0.1679	2.13E-73
Mg-C	0.0021	3.7629	0.9260	0.3694	2.96E-19	0.0204	3.08E-129
S-A	0.0063	4.3700	1.3066	0.6900	6.40E-08	0.1911	6.01E-08
S-C	0.0015	4.3800	1.2760	0.5470	4.75E-12	0.0307	2.34E-11
Fe-A	0.0010	7.0500	0.2013	0.4963	2.72E-37	0.1023	0.2353
Fe-C	0.0007	4.2090	0.2164	0.3608	4.00E-33	0.0222	0.3012
Mn-A	0.0000	1.1415	0.0085	0.0585	1.17E-39	0.0224	0.7351
Mn-C	0.0001	0.6815	0.0077	0.0363	4.85E-39	0.0101	0.7021
Na-A	0.0025	4.7840	0.7881	0.5499	1.24E-21	0.0520	6.06E-22
Na-C	0.0011	12.2300	0.8342	0.7908	8.93E-31	0.0119	9.15E-12

^a^Sharipo test was used for normality test; ^b^High and normal date-sample student’s t-test at *p* value < 0.01.

Pearson’s correlation coefficients (*r*^*2*^) were used to examine the natural variation within the population of quantified traits between normal and high Mg^2+^ conditions (Table 2). The RG and SG showed positive correlations between normal and high Mg^2+^ treatments (*r*^*2*^ = 0.53) (Table 2). A weak correlation was observed for nutrient traits, especially Ca, Fe and Mg concentrations between normal and high Mg^2+^ treatments (*r*^*2*^ < 0.2), suggesting different physiological functions of nutrients under different Mg^2+^ supplies.

Cluster of Pearson’s correlation coefficient between phenotypes (Heat map) showed a negative correlation between Ca-C-H, Fe-C-H and Na-C-H and Mg-C-H (Fig. 2C), suggesting that Mg^2+^ would compete for uptake with Ca^2+^, Fe^2+^ and Na^2+^ in most *Arabidopsis* accessions when supplied with high Mg^2+^. Increased Mg^2+^ absorption would decrease the uptake of these ions and hence plant growth. However, Mg-A, Mg-C, S-A, S-C, K-A and K-C were positively correlated only under high Mg^2+^ (Fig. 2C), indicating a common mechanism or genetic pathway between Mg^2+^, S and K under high Mg^2+^ (Fig. 2C). In contrast, there were highly significant positive correlations between PR, RW, LR, FW as well as S-A, Mg-A and K-A of the phenotypes under normal but not high Mg^2+^ supply (Fig. 2C).

### GWAS analysis for nutrient metabolism in response to high Mg^2+^

**Figure 3:**
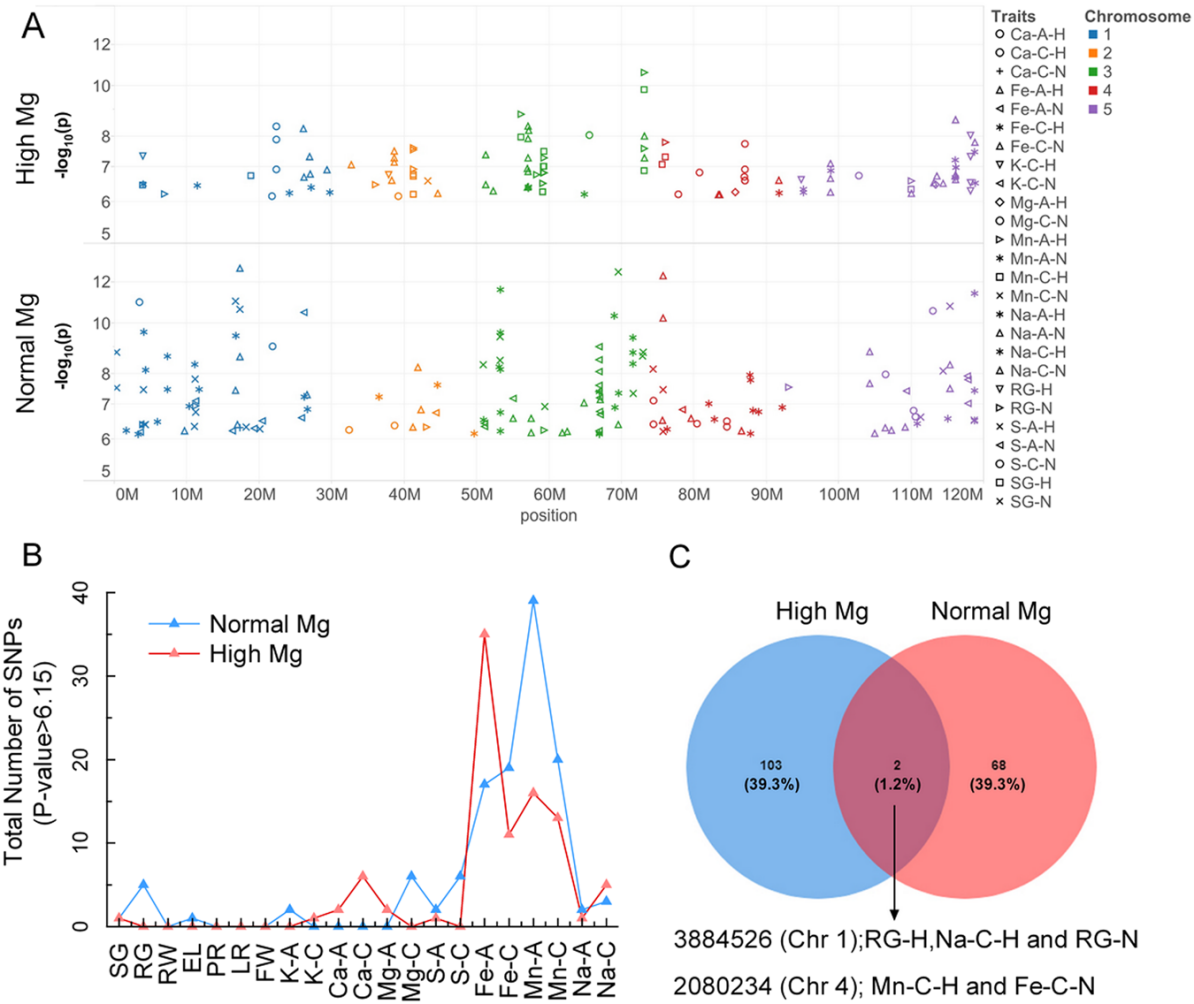
GWASs for 21 traits under normal (1,000 µM) and high Mg^2+^ (10,000 µM) conditions. The x-axis is the chromosomal coordinates for *Arabidopsis*, the y-axis represents *p*-value. The GWASs hits observed at high Mg^2+^ supply were presented on the top; the hits observed at normal Mg^2+^ were represented at the bottom panel (**A**). GWAS hits and chromosome were represented by different symbols and coloured, respectively. GWAS hits were shared between normal and high Mg^2+^ were ordered by their longitudinal (x-axis) position. A SNP GWAS hit was defined as the one had −log10(*p*) > 6.15. (**B**) Total number of traits that were scared for SNPs with log10(*p*) > 6.15. The x-axis represents scared traits for *Arabidopsis*, the y-axis is the *p*-value. Blue and Red lines represent normal and high Mg^2+^, respectively. (**C**) Overlap between total number SNP hits for 21 traits at normal and high Mg^2+^. Venn diagrams display overlap between SNP number at normal Mg^2+^ (blue) and high Mg^2+^ (red).

The phenotypic data collected from normal and high-Mg^2+^ conditions (Supporting Information Table S1) were used as an input for genome-wide association study (GWAS). All SNPs were converted to homozygote or heterozygote according to the genotype, and tri-allelic or tetra-allelic SNPs were removed. Candidate loci were selected based on the minor allele frequency, LOD score and number of significantly associated SNPs within one locus. Using −log_10_(*p*) > 6.15 as the nominal genome-wide significance threshold, the GWASs had 109 hits in total, 32, 10, 25, 18 and 24 located on chromosomes (Chr) 1, 2, 3, 4, and 5, respectively, under normal Mg^2+^, and 79 hits in total, 14, 13, 18, 10 and 19 located on chromosomes 1, 2, 3, 4, and 5, respectively, under high Mg^2+^ (Fig. 3A,B). There were 2 shared hits between normal and high Mg^2+^ supplies (Fig. 3C).

Under normal Mg^2+^ supply, 41 and 22 loci were associated with Mn absorption (Mn-A-N) and concentration (Mn-C-N), and accounted for 7.47% and 2.67% of the total phenotypic variance, respectively. 17 and 21 loci were associated with Fe absorption (Fe-A-N) and concentration (Fe-C-N), and accounted for 3.60% and 5.04% of total phenotypic variance individually (Fig. 3B, Supporting Information Table S3). In comparison, under high Mg^2+^ supply, 16 and 13 loci were associated with Mn absorption (Mn-A-H) and concentration (Mn-C-H), and accounted for 2.93% and 12.04% of total phenotypic variance individually. Thirty three and 11 loci were associated with Fe absorption (Fe-A-H) and Fe concentration (Fe-C-H), which accounted for only 5.07% and 2.60% of total phenotypic variance individually (Fig. 3B, Supporting Information Table S2). The GWAS analysis confirmed that only 2 common loci were detected differed between normal Mg^2+^ and high Mg^2+^ (Fig. 3C).

Single nucleotide polymorphisms (SNP) 1_3884526 (Chromosome 1, 3884526 bp) was found to associate with SG-H, RG-H, RG-N and Na-C-N. It had a Minor allele frequency (MAF) of 0.0633, and is within the *OST3A*, a gene related to oligosaccharyl transferase complex/magnesium transporter family (Supporting Information Table S2). SNP 4_2080234 was associated with Mn-C-H and Fe-C-N. It had a MAF of 0.050 and is within *AT4G042960* and *AT4G04293*, both transposable element genes.

No significant associations were observed for the Mg^2+^ (Mg-A) and Ca absorption (Ca-A) under normal Mg^2+^, but 2 loci were identified to associate with these traits under high Mg^2+^. Furthermore, no significant associations were found for Mg^2+^ (Mg-C) and S concentration (S-C) under high Mg^2+^, whereas 7 and 6 loci were identified to associate with these traits under normal Mg^2+^ (Fig. 3B). No significant associations for K absorption (K-A) were identified under normal and high Mg^2+^.

**Figure 4:**
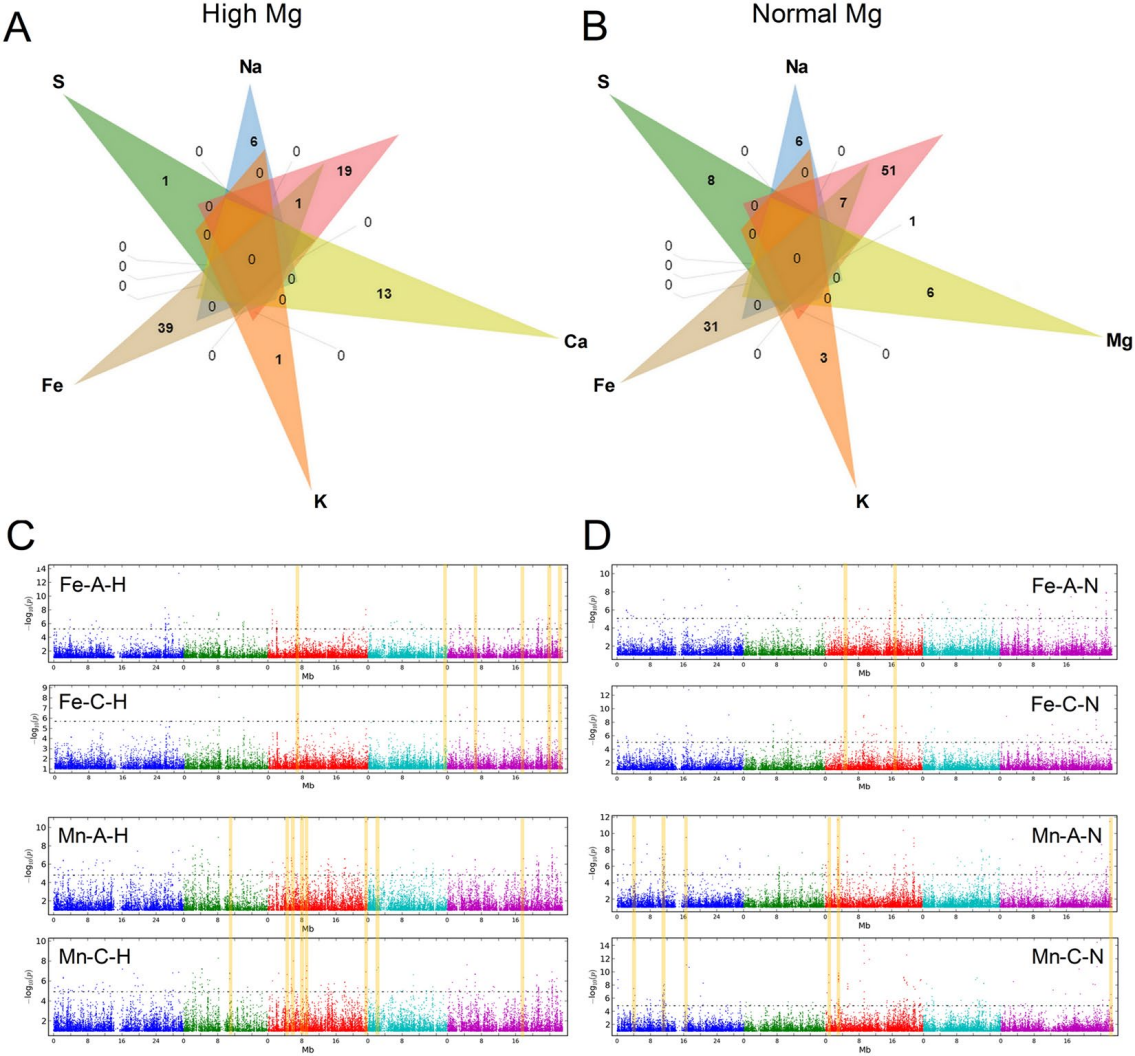
Venn diagrams representing differentially nutrient (including absorption and concentration) GWAS hits was defined as the one had −log10(*p*) > 6.15 in high Mg^2+^ (**A**) and normal Mg^2+^ (**B**) of *Arabidopsis*. Each list in the venn diagram denotes by a transparent shape and overlaps shape indicates elements shared between lists or more often the corresponding counts. Four lists of input data of Fe-A, Fe-C, Mn-A and Mn-C were highlighted for both high Mg^2+^ and normal Mg^2+^ in green, blue, pink and yellow, respectively. Genome-wide association mapping results for the absorption and concentration of Fe and Mn in *Arabidopsis* under high Mg^2+^ (**C**) and normal Mg^2+^. (**D**) The ‘Manhattan setting’ plate for customized chromosome color, fixed pixel size, or dynamic pixel size proportional to *p*-values in Manhattan plot. The 5 chromosomes are represented by different shades of gray with the x-axis as the physical position. The highlighted positions represent the physical positions of the regions sharing common SNP between absorption and concentration of Fe and Mn.

We speculated that the absorption and concentration of nutrients should share many candidate loci associated with the same element under the same Mg^2+^ level. However, this was not the case in our data set, as no common candidate loci were observed to associate with absorption and concentrations of K, Ca, Mg, S and Na under either normal or high Mg^2+^ (Supporting Information Table S2). Though common loci were found between the absorption and concentration of Fe or Mn in plants grown under both normal and high Mg^2+^, these only accounted for a small part of the variance in attitudes. While six common loci were detected between the traits of Fe-A-H and Fe-C-H, only 2 common SNPs were for Fe-A-N and Fe-C-N. Likewise, 10 common loci were associated with Mn-A-H and Mn-C-H and 6 common loci associated with Mn-A-N and Mn-C-N (Fig. 4A,B). Furthermore, manhattan plot showed the detailed distribution of the GWAS hits for Fe and Mn (Fig. 4C,D).

### Characterization of *Atntrl* mutants in response to high Mg^2+^ treatment

**Figure 5:**
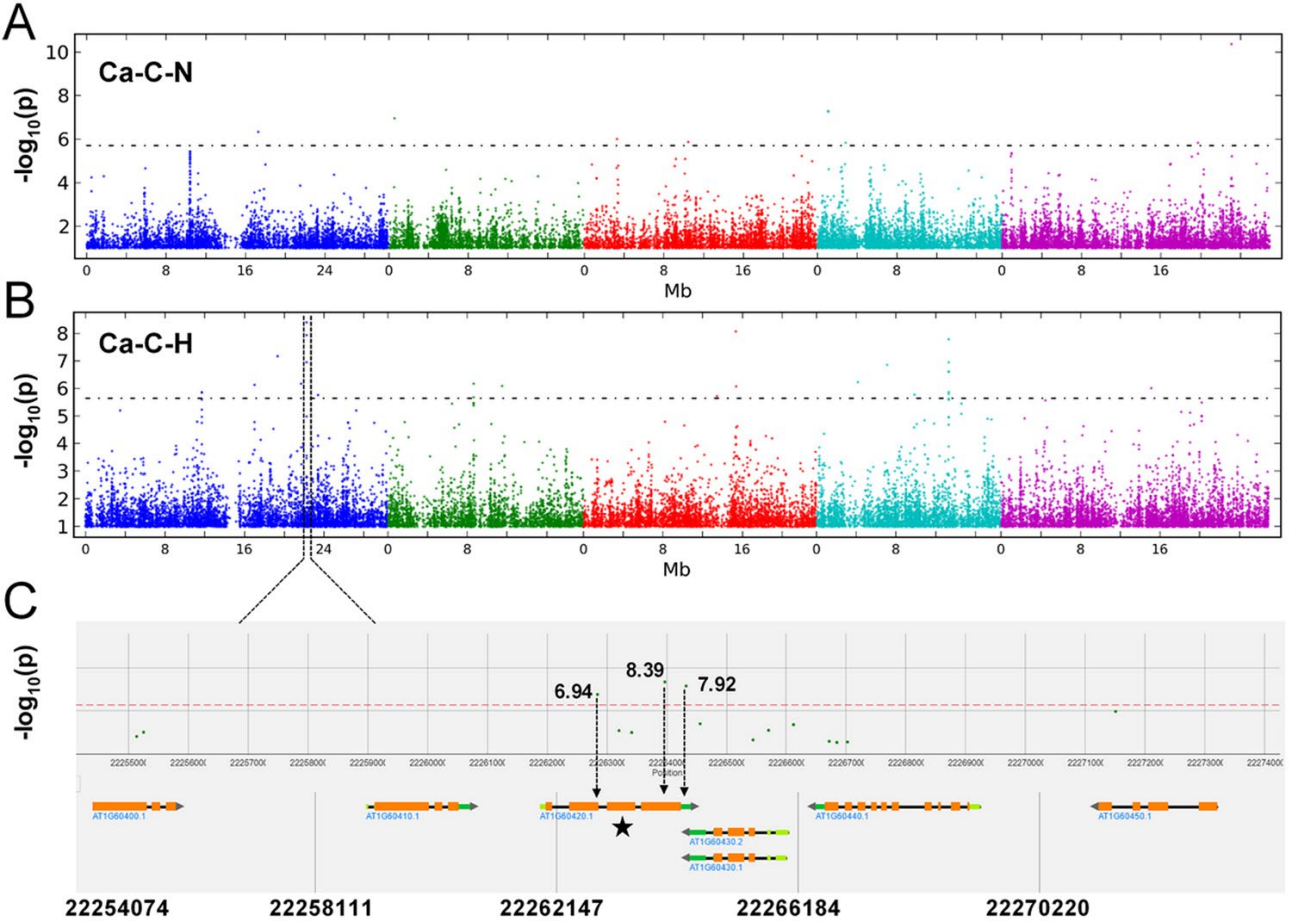
Manhattan plot illustrating the GWAS mapping of the Ca concentration in 388 *Arabidopsis* accessions grown under supply of normal (1,000 µM) (**A**) and high Mg^2+^ (10,000 µM). (**B**) Chromosomes are shown in different colors. (**C**) Detailed plot of the region shown in the red box in (**B**). The positions on the chromosome are on the x axis and the score on the y axis. The dots in the scatterplot represent SNPs. A horizontal dashed line shows the 5% threshold.

GWAS is conducted to identify causative/predictive factors for a given trait, or to determine aspects of the genetic architecture of the trait (i.e. the number of loci that contribute to the phenotype)^41^. A single strong peak of SNPs 22262848 between position of 22262147 and 22266184 bp on chromosome, which is associated with Ca concentration, was identified under high but not normal Mg^2+^ (Fig. 5A,B). This SNP accounted for 0.326% of the total variance of an estimated 2.20% of the total variance in Ca concentration under high Mg^2+^. It contained three neighboring significant SNP hits (−log_10_(*p*) > 6.9) corresponding to only a candidate gene *At1g60420* (*AtNRX1*) (Fig. 5C), and was *calcium locus CD-domain containing protein*.

**Figure 6:**
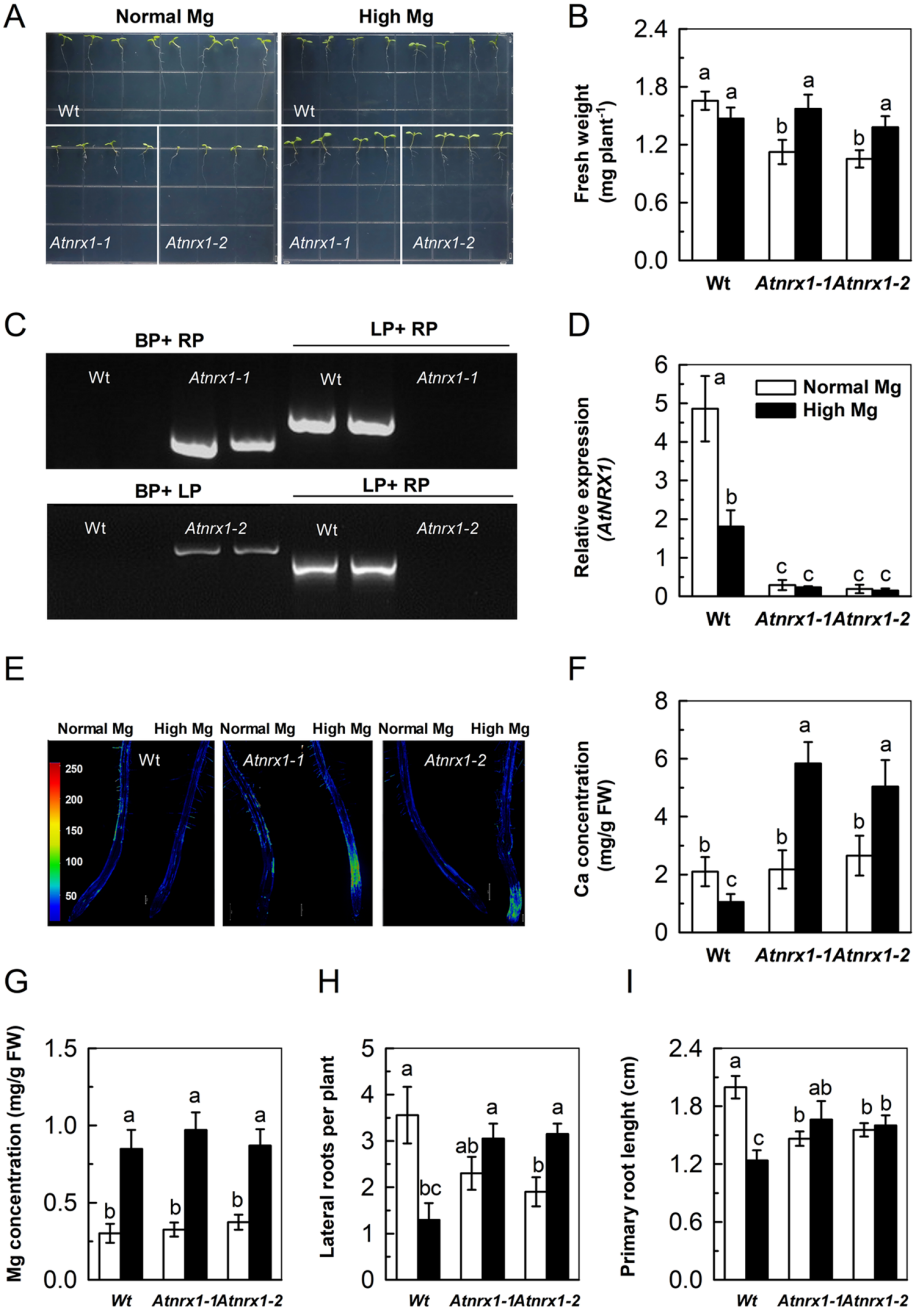
Validation and physiological phenotypes of *Atnrx1* mutant. Image of mutant *Atnrx1-1* and *Atnrx1-2* and wild plants in media with normal or high Mg^2+^ (**A**); results of T-DNA insertion mutant *Atnrx1-1* and *Atnrx1-2* by PCR with gene-specific primers LP, RP and LB (**B**); relative expression levels (**C**); fresh weight (**D**); confocal images of cytosolic Ca^2+^ concentration distribution (Fluo-4/AM imaging) during the initiation and tip growth of root hairs (**E**); concentrations of Ca (**F**); concentrations of Mg^2+^ (**G**); number of lateral root per plant (**H**) and the length of primary root (I) of wild-type and *Atnrx1* mutant (8-day-old seedlings) grown at supply of normal (1,000 µM) and high Mg^2+^ (10,000 µM). Levels of cytosolic Ca^2+^ concentration have been color-coded according to the inset scale (Quantitative values under the scale in nM). Bar = 200 *μ*m applies to all photographs in D. The images are representative of at least three independent experiments. Data are means ± SD (n = 5). Means followed by a common letter within a root segment are not significantly different at *P* < 0.05. FW, fresh weight.

To validate the GWAS results in the present study, the above putative significant candidate gene was determined through a collection of well-characterized mutants (*Atnrx1-1* and *Atnrx1-2*) and physiological and morphological comparisons in mutants and wild-types. Before this, we further verified that the mutant stocks were homozygous T-DNA insertion lines, by PCR with gene-specific primers LP, RP and LB. As expected, the results confirmed that both *Atnrx1-1* and *Atnrx1-2* were only mutation in *At1g60420* (Fig. 6A). Moreover, the expression of *AT1G60420* were greatly reduced in two mutants under both normal and high Mg^2+^ conditions as compared to wildtype (Fig. 6B). Compared with normal Mg^2+^, high Mg^2+^ increased the fresh weight of *Atnrx1-1* and *Atnrx1-2* but did not affect that of wildtype. It is worth mentioning that compared with wildtype, fresh weight of two mutants was not changed under high Mg^2+^ but was reduced under normal Mg^2+^ (Fig. 6C). It is suggested that high Mg^2+^ reduced wildtype growth but had little effect on the growth of *Atnrx1* mutant.

As shown in Fig. 6D, a tip-focused [Ca^2+^]_c_ gradient was formed during the initiation and elongation of roots of both wildtype and mutants, and such a tip-focused gradient of [Ca^2+^]_c_ was weak under normal Mg^2+^. In contrast, under high Mg^2+^, *Atnrx1-1* and *Atnrx1-2* but not wildtype formed a strong tip-focused [Ca^2+^]c gradient in the initiation and elongation of root of the mutant (Fig. 6D). Furthermore, high Mg^2+^ greatly enhanced concentration of Ca in *Atnrx1-1* and *Atnrx1-2* but decreased it in wildtype as compared with normal Mg^2+^ (Fig. 6E). Meanwhile, when compared with wildtype, Ca concentration of high Mg-fed *Atnrx1-1* and *Atnrx1-2* mutants was greater while that of normal Mg-fed *Atnrx1-1* and *Atnrx1-2* mutants was similar (Fig. 6E). As expected, high Mg^2+^ enhanced Mg concentrations in both mutants and wildtype (Fig. 6F).

Compared with the wildtype, both *Atnrx1-1* and *Atnrx1-2* had shorter primary root and less number of lateral roots under normal Mg^2+^ but had greater length of primary roots and a similar number of lateral roots under high Mg^2+^ (Fig. 6G,H). Under high Mg^2+^, there is no significant difference in K, Fe, Mn and S concentrations in plants between the two mutants and wildtype, but the mutant had a higher Na content (Data not shown). These results indicate that *AtNRX1* is a special and critical negative gene regulating Ca uptake and probably the plant growth under high Mg^2+^ stress.

## Discussion

To adapt to the constantly high availability of Mg^2+^ in the environment, plants have evolved mechanisms of reduced ion uptake and/or sequestering excessive ions in the vacuole or developing higher Mg^2+^ requirement for the maximum growth, which finally improves the tolerance to the stress of certain elements. By linking the mapping with reverse genetic experiments, we identified the causal gene and established that polymorphisms at *AtNRX1* were the major genetic determinant for the variation in Ca concentration in this global *A. thaliana* population (Fig. 6). *Atnrx1* mutants have most of the phenotypes that would associate with the tolerance to serpentine soils, including survival in solutions with a low Ca^2+^: Mg^2+^ ratio; requirement for a high concentration of Mg^2+^ for maximum growth; greater biomass and more lateral roots, reduced leaf tissue concentration of Mg; and poor growth performance on ‘normal’ levels of Mg^2+^.

### Shoot and root morphology variation in response to high Mg^2+^

In order to understand molecular mechanisms of plant adaptation to high Mg^2+^ stress, we grew 388 wild *Arabidopsis* accessions at normal and high Mg^2+^ concentrations, and observed a substantial quantitative variation in plant growth between these accessions (Fig. 1; Table 1). We also showed a diversity in shoot and root morphology across two levels of Mg^2+^ supply and among these natural accessions. High Mg^2+^ supply resulted in an overall reduction in primary root length, lateral root number, epicotyl length and fresh weight but its effect on the rosette width varied among the accessions (Fig. 2; Table 2). The phenotypic data indicate that *Arabidopsis thaliana* has evolved some capacities to adapt to high Mg^2+^ stress and that the number of lateral roots of seedlings is a suitable index of high-Mg^2+^ responses.

By using the phenotypic data of 388 *Arabidopsis* accessions (Supporting Information Table S1) and GWAS, this study showed that a great extent of variation in the length of primary and lateral roots, rosette growth and hypocotyl length among the accessions tested was strongly Mg-dependent. As we known, the growth of primary and lateral roots, hypocotyl and rosette is determined by a number of extrinsic and intrinsic factors in addition to Mg^2+^ supply. The significant associations with the leaf or root growth-related phenotypes according to −log_10_(*p*) > 6.15 reflects the high complexity of the underlying genetic architecture or that these traits were not influenced by any of the SNPs alone. This notion is supported by the presence of obvious sub-threshold peaks associated with shoot and root morphology under both normal and high Mg^2+^ supply. Meanwhile, the low heritability of investigated traits in the present study suggested a weak genetic component determining the observed phenotypic variability. Hopefully, it is known that many traits of *Arabidopsis* are polygenic with small effect size, and that increasing the sample size would improve the power to recover meaningful associations^42^. Therefore, increasing sampling size was likely to underestimate the range natural variation in photomorphogenic responses or growth-related pathways and processes that strongly interact with the high Mg^2+^ stress.

### *Arabidopsis* showed remarkable natural genotypic variation for ionomics under high Mg^2+^

The present study showed that most of the SNPs were derived from the nutrient concentration and uptake, SNPs differed between normal and high Mg^2+^, the effective number of SNPs (−log_10_(*p*) > 6.15) was also different (Fig. 3; Supporting Information Table S2). For example, positive SNPs associated with Ca-A and Mg-A were located only under high Mg^2+^ (Fig. 3B). Likewise, no significant associations were observed for either Mg-C and S-C under high Mg^2+^ but seven and six significant SNPs were associated with them under normal Mg^2+^ (Fig. 3), indicating that different genetic systems were responsible for Mg^2+^ and S uptake under normal and high Mg^2+^. Meanwhile, the SNP significance tests showed that different traits were generally associated with different genes or gene regions, but many of the traits also shared common genes or gene regions. The significant score >10 association was for the SNP on Mn under high Mg^2+^ in a narrow region of chr3, where multiple SNPs were associated with different traits in Chr1, 3, 4 and 5 under normal Mg^2+^ (Fig. 3).

Though not all cases, the chromosomal regions harbouring the significantly associated SNPs are coincided with the location of the gene(s) co-expressed with known-function genes that control these traits. Of the 105 loci under normal Mg^2+^, the majority of the loci were associated with Mn-A and Mn-C then with Fe-A and Fe-C. Among these, SNP 1_3953597, which is within the SS3, a gene encoding a starch synthase, is co-expressed with *SPLICING ENDONUCLEASE 1 (SEN1)*^43^. Likewise, SNP 3_19424421 of *p*-value 12.55, associated with Mn-C under the normal Mg^2+^ condition, had a MAF of 0.058 and was within gene *AT3G52380 CHLOROPLAST RNA-BINDING PROTEIN 33* (*CP33*) that is co-expressed with *SEN1*. *SEN1* gene was reported encoding an integral membrane protein homologous to *Glycine max* nodulin-21, and also to *CCC1*, a vacuolar Fe/Mn transporter of Saccharomyces cerevisiae of *Arabidopsis*^41^. Besides, SNP 1_210424, associated with Fe-C, had a MAF of 0.055 and was within gene *FRO2* which encodes the low-Fe-inducible ferric chelate reductase. It is suggested to be the major Fe(III) chelate reductase in *Arabidopsis*^44^. The most SNP hits under high Mg^2+^ were identified associations with Fe-A and Fe-C and then Mn-A and Mn-N. SNP 1_26930918 had a MAF of 0.074 and was located within *AT1G71480* and *AT2G33410* genes which are involved in nuclear transport factor 2 family gene, and are co-expressed with Fe transporter gene *FERRIC REDUCTION OXIDASE 6 (FRO6)* and *7 (FRO7)*^45^ and *1 (FRO1)*^46^. Furthermore, SNP 2890675, associated with Fe-C under the high Mg^2+^ on Chromosome 5, had a MAF of 0.074 and was located within *AT5G09310* gene and co-expressed with Fe transporter gene *FRO1*^46^. Likewise, SNP 3_6977829 of *P*-value 8.40, associated with Fe-A, within gene *AT3G20015* (*ASPG2*), and was co-expressed with *H*( + )-*ATPASE 2 (AHA2)*, a main actor in Fe uptake and signaling^47^.

In addition, SNP 4_772594 associated with Mg + C under the normal Mg^2+^, had a MAF of 0.079 and was located within *AT4G01800 ALBINO OR GLASSY YELLOW 1* (*AGY1*) gene which was co-expressed with *MAGNESIUM CHELATASE I2 (CHLI2)* while *CHLI2* regulates the function of Mg^2+^ chelatase^48^. It should be mentioned that SNP 1_21762851 of *P*-value 9.05, associated with S-C under the normal Mg^2+^, had a MAF of 0.090 and contained only one gene, *AT1G51980*, which encodes an insulinase and is co-expressed with *SULFATE TRANSPORTER 1;2 (SULTR1;2)* that is involved in sulfur metabolism^49^. As discussed above, changing the Mg^2+^ availability in the soil would alter absorption and utilization of other elements, and transporters, channels, or/and the genes that encode and regulate them in *Arabidopsis*. However, a few of the genes in proximity of the most significant associations were among the major known regulators of ion uptake or transport, highlighting the potential of GWAS to identify previously unknown regulators^50^.

Changes in nutrient availability influence plant vegetative growth and phenotypic traits of *A. thaliana*, but the effect may vary among populations or genotypes^51–53^. Decreases in nutrient availability result in phenotypic responses (such as smaller leaf areas, shorter primary root and reduced lateral root formation) that vary across *A. thaliana* genotypes.

### A novel candidate gene *AtNRX1* negatively regulates Ca concentration at high Mg^2+^ supply

Calcium is an essential plant macronutrient with key structural and signaling roles. Calcium ions (Ca^2+^) act as an osmoticum within vacuoles, a stabilizing element in the membranes, a strengthening agent in cell walls, and a secondary messenger for a multitude of signals^54–56^. By using GWA mapping on the 388 *A. thaliana* accessions, we successfully identified a single strong peak of SNP 1_22263976/22264331/22262848 (Chromosome 1, 22263976 bp) associated with Ca concentration under high Mg^2+^ (Fig. 5). Mutants of *Atnrx1-1* and *Atnrx1-2* supplied with high Mg^2+^ had higher cytosolic Ca^2+^ concentrations ([Ca^2+^]_c_) during root elongation, meanwhile produced higher biomass and more lateral roots as compared with wildtype (Fig. 6). This suggested that the increase in Ca concentration in mutants of *Atnrx1-1* and *Atnrx1-2* by high Mg^2+^ cannot be attributed to increased biomass or photosynthesis alone, but also to the improved Ca nutrition of the plants through morphological, physiological and molecular responses to high Mg^2+^.

It has been known that *AtNRX1* is involved in DC1 domain-containing protein, which is co-expressed with calcineurin B-like Ca sensor 1–protein kinase gene *CBL-INTERACTING PROTEIN KINASE 23 (CIPK23)*^57,58^. Study of Tang *et al*.^59^ showed that tonoplast CBL–CIPK calcium signaling network regulated ion homeostasis and vacuolar sequestration of Mg^2+^, thereby protecting plants from Mg^2+^ toxicity^59,60^. Besides, loss-of-function *cax1* (gene encoding vacuolar H^+^/Ca^2+^) mutations could produce phenotypes characteristic of plants adapted to serpentine^8^.

This locus has a MAF of 0.0712 and is within the *AT1G60420* (*AtNRX1)*, a gene involved in catalytic activity of NADP-NADPH and disulfide-reductase activity^61^. In special cases, toxic ROS molecules are largely accumulated under excess stresses and diverse enzymes played as ROS scavengers. *Arabidopsis* contains 3 NADPH-dependent thioredoxin reductases (NTRs) for scavenging ROS and Nucleoredoxins (NRX) are potential nuclear TRX found in most eukaryotic organisms^61^. Recently, excellent research presents evidence that activity of the plant stresses-inducible TRX superfamily member, *NRX1* is necessary for the integrity of antioxidant systems^62^. Meanwhile, it is the fact that many publications indicated ROS produced by NADPH oxidase activates Ca^2+^ channels at the plasma membrane, leading to an increase in Ca^2+^ concentration and a tip-focused Ca^2+^ concentration gradient and subsequent signaling inherent to plant growth^32,63–66^. Withal, our previous study also showed that high Mg^2+^ could inactivate NADPH and the production of ROS^27^ probably through regulating ABA–DELLA signaling^28,67^.

To sum up, it is likely that under high Mg, the wild-type plant is associated with cellular accumulation of ROS and redox imbalance, which triggers class of antioxidant enzymes activation and subsequently results in a decrease of intracellular ROS. ROS restrains ion channels and Ca^2+^ concentration; While in the *nrx1* mutants, ROS could not be effectively removed without protection of *NRX1* due to *NRX1* mutation, which leads to ROS accumulation. Then ROS activate Ca^2+^-channels at the plasma membrane and promote cytosolic Ca^2+^ concentration. When both wild-type and *nrx1* mutants under normal Mg conditions, the cell is normally controlled with H_2_O_2_ and redox balance, thus the protective effect of *NRX1* gene is not prominent, or maybe even little dedication.

Notably, Mg^2+^ concentrations in *Atnrx1-1* and *Atnrx1-2* mutants was increased compared to that in the wildtype plants under high Mg^2+^ supply. This implied that *nrx1* mutants required for a high concentration of Mg^2+^for the maximum growth associating with tolerance to high Mg^2+^ soils. The *AtNRX1* gene is co-expressed with *MAGNESIUM TRANSPORTER 4 (MRS2-3)*, a transport gene related to Mg^2+^ nutrition^68^. These indicate that Mg^2+^ transporter genes have some interactions with *AtNRX1*. However, there is no significant difference in K, Fe, Mn and S concentration in plants between the two mutants and wildtype, but the mutant had a higher Na content (Data not shown). These was consistent with other study showed that the *cbl2 cbl3* double mutant was hypersensitive to a handful cations but not to Na^+^ ^59^. Further work will be directed to identify other key components in this novel of NRX-signaling pathway in regulation of Ca uptake and how the positive effects of *AtNRX1* can be maximized through plant adaptive strategies to improve the tolerance to high Mg^2+^.

To sum up, our study suggests that *AtNRX1* was a critical gene negatively regulating Ca uptake and probably improving the plant tolerance to high Mg^2+^ soils. The discovery of the functions of *AtNRX1* gene helps to breed/select crops that can adapt to high-Mg^2+^ soils such as serpentine soils (high Mg^2+^: Ca) and those in semi-arid regions or even serpentine or Mars soils with high levels of magnesium sulfate. Also, new genes associated with natural variation in *A. thaliana* can be used for future bioengineering.

## Electronic supplementary material

Supplemental infomation

Table S1

Table S2

Table S3
