# Supplementary material for: Natural variation among Arabidopsis thaliana accessions in tolerance to high magnesium supply
Source: Sci Rep. 2018 Sep 11;8:13640. doi: 10.1038/s41598-018-31950-0 (PMC6134027; doi:10.1038/s41598-018-31950-0)
Supplement: Supplementary file 1 — Supplemental infomation [file 41598_2018_31950_MOESM1_ESM.doc]

**Natural variation among *Arabidopsis thaliana* accessions in tolerance to high magnesium supply**

**Yaofang Niu^1*^,** **Ping** **Chen^1*^, Yu Zhang^1*^,** **Zhongwei Wang^1^, Shikai Hu^1^, Gulei Jin^2^, Caixian Tang^3^,****Longbiao Guo ^1^**^†^

**Additional Information**

**Supplementary Table. S1.** List of data of 42 traits under normal (1,000 µM) and high Mg (10,000 µM) that were used for GWA analysis.

**Supplementary Table. S2.** List of associated SNPs and candidate genes within 5 kb up- and down-stream of the identified SNP under normal (1,000 µM) and high Mg (10,000 µM).

**Supplementary Table. S3.** List of the putative candidate genes corresponding to each SNP under normal (1,000 µM) and high Mg (10,000 µM).
